# Supplementary material for: Evolution of default genetic control mechanisms
Source: PLoS One. 2021 May 13;16(5):e0251568. doi: 10.1371/journal.pone.0251568 (PMC8118313; doi:10.1371/journal.pone.0251568)
Supplement: S1 File — (PDF) [file pone.0251568.s001.pdf]

Evolution of default genetic control mechanisms

Appendix 1. Energy required for protein synthesis

William Bains, Enrico Borriello, Dirk Schulze-Makuch

Lane and Martin state (1) that the majority of energy in a cell goes into protein synthesis. Their citation for this is a table in a textbook (2), which refers to heterotrophic bacteria growing under unlimited nutrient (i.e. non-natural) conditions, usually in a lab. or factory where conditions have been optimized for maximum growth (reviewed in (3)). This did not seem like a satisfactory basis for evolutionary theory [1], so we compiled the measured energy used by a wide range of cells and organisms under conditions of maximal growth. Obviously, under conditions of sub-maximal growth (which are conditions experienced by almost all organisms almost all the time) the energy use for protein synthesis will be lower.

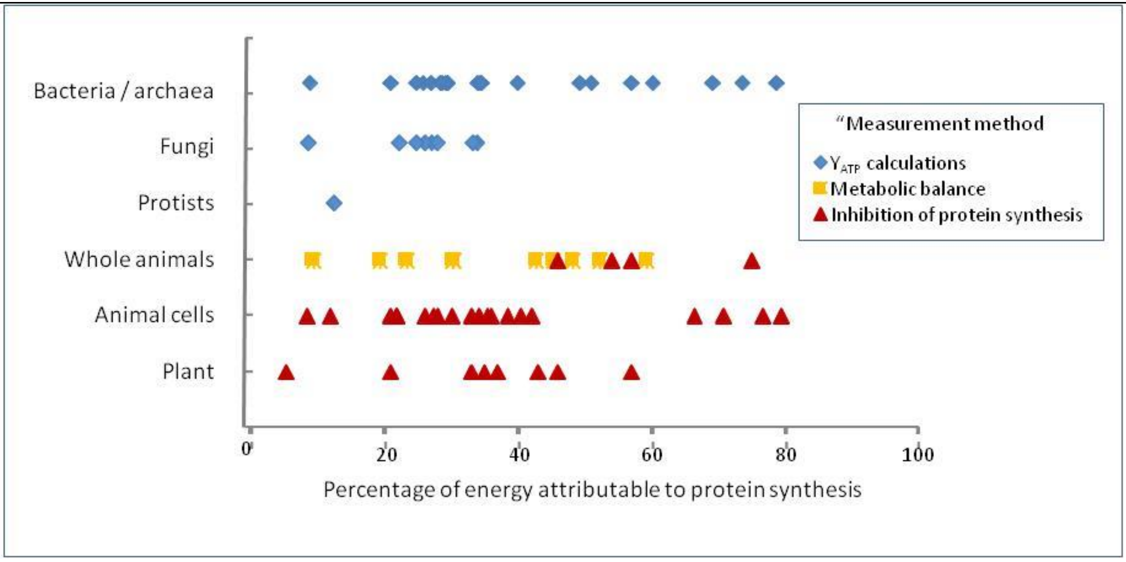

Figure 1: Fraction of energy consumption used for protein synthesis
Y axis – category of organism. X axis – fraction of energy consumption put into protein synthesis under conditions of maximal growth for each organism. See text for details.

In summary, only rarely is the fraction of the energy of organisms or cells growing at a maximal rate found to be above 50% of the energy used by a cell.

---

[1] By analogy to this argument, by taking an extreme value as a representative value, we could prove that the human brain consumes less than 5% of a human's calorie requirement, in line with most other vertebrates. The brain requires 280-300 kCal/day, an adult male consumes 6000 kCal/day (based on early 20th Century Antarctic explorers : See reference (5)) therefore the human brain only uses 5% of the body's energy. The fallacy is equating the normal population with the highly abnormal and contrived environment of explorers trying to walk to the South Pole. Equally contrived is equating the growth of *E.coli* on LB Broth with its normal evolution in the gut.

There are four approaches to estimating the fraction of energy that is used by a cell or an organism to synthesise proteins.

The first approach, cited by (1), conducts a theoretical calculation based on the metabolic pathways leading to amino acids, the energetic cost of protein polymerization, and the fraction of the cell biomass that is protein. This leads to a maximum possible partition of energy into protein production, typically ~80% for heterotrophic bacteria.

The second approach is to measure metabolite flux into and out of a cell, calculate the energy generated from different metabolic pathways involved in uptake and transformation of substrates, and convert this to an equivalent of ATP molecules for comparison (6, 7). The fraction of this being devoted to protein synthesis (called Y_{ATP}) can then be calculated from the amount of protein synthesised and the theoretical amount of ATP needed to synthesise protein, as above.

The other two methods are less direct, but are suitable for organisms where i) the rate of adaptation of metabolism to perturbations is slow compared to the timescale of the experiment, and ii) metabolic flexibility is more limited than in bacteria. The first is to inhibit protein synthesis with a specific inhibitor (typically cyclohexamide), and measure the difference in energy capture (usually respiration, although the method also works for anaerobic organisms) between inhibited and non-inhibited state. This under-estimates the energy of protein synthesis (as protein synthesis is rarely completely inhibited – specifically, mitochondrial protein synthesis is not blocked), but also over-estimates the energy used in protein synthesis (as other cell processes are rapidly down-regulated if protein synthesis is inhibited (8)). The fourth, which is suitable to animals in particular, is to measure food intake, waste products and protein gain, and compute from standard tables of the energy that an animal can derive from food what fraction of that energy has been used to synthesise protein. This obviously varies greatly with how much food the animal is given (which is the point).

Literature values for all experimental measurements were sought be keyword search, and by following citations to and references in identified papers. There are regrettably very few studies on protists, and those entirely confined to the protists in the rumen fluid of farmed ruminant animals. A wide range of protist respiration rates have been measured (reviewed in (9)), but apportionment of energy to growth and to maintenance not specified. Studies of photosynthetic organisms are problematic as total cell or organism energy is usually calculated from the amount of biomass gained, assuming that the researcher knows the amount of energy needed to create a gram of biomass – calculating the efficiency of forming biomass from this energy value would therefore be circular. The few examples from plants are therefore from dark respiration in leaves or from non-photosynthetic tissues such as flower petals.

The three tables below list the values shown in Figure 1 by method of measurement. Y_{ATP} values were converted to fractional energy of protein synthesis assuming a maximum possible Y_{ATP} of 30 for E.coli (10, 11), a protein composition of 52% of the dry cell weight for bacterial cells (11, 12), 40% for fungal cells (13, 14) and 47% (as an intermediate value) for other cells. Where a range of values are given in a paper for a range of growth conditions, the highest value is cited here (obviously the lowest value for any organism is 0).

Metabolic input/output analysis method

Kingdom	Species	Test subject	Feeding regime	Fraction / value	Ref
Animalia	juvenile African catfish C. gariepinus	Whole animals	Balanced	30%	(15)
Animalia	Sydney rock oysters (Saccostrea commercialis)	Whole animals	Minimum growth	9%	(16)
Animalia	Sydney rock oysters (Saccostrea commercialis)	Whole animals	Maximum growth	19%	(16)
Animalia	toad, Bufo bufo	Whole animals	Maximum growth	52%	(17)
Animalia	toad, Bufo bufo	Whole animals	Baseline growth	48%	(17)
Animalia	Calanus finmarchicus	Whole animal	Maximal	52%	(18)
Animalia	Acartia tonsa	Whole animal	Maximal	42.5%	(18)
Animalia	Beef cattle	Adult	Balanced	23%	(19)
Animalia	Mytilus edulis	Adult	Maximal	30%	(20)
Animalia	Rat	Adult	Maximum	59%	(21)
Animalia	Rat	Adult	Balanced	45%	(21)

Protein synthesis inhibition

Kingdom	Species	Test system	Growth conditions	Fraction energy in protein synthesis	Reference
Plantae	Solanum tuberosum	Cell suspension	N/A	33%	(22)
Plantae	Phaseolus vulgaris	Growing leaf	Dark reaction respiration	37%	(22)
Plantae	Phaseolus vulgaris	Mature leaf	Dark reaction respiration	21%	(22)
Plantae	Triticum aestivum	Mature leaf	Dark reaction respiration	35%	(23)
Plantae	Petunia hybrida	Petals (20oC)	Endogenous respiration	43%	(24)

Plantae	Petunia hybrida	Petals (25oC)	Endogenous respiration	46%	(24)
Plantae	Petunia ¥ hybrida	Petals (35oC)	Endogenous respiration	5.4%	(24)
Animalia	sea urchin Lytechinus pictus	Larva	Maximal	75%	(25)
Animalia	sea urchin Lytechinus pictus	Whole organisms (embryos)	N/A	54%	(25)
Animalia	Antarctic limpet, Nacella concinna	Adult (0oC)	Balanced	46%	(26)
Animalia	Antarctic limpet, Nacella concinna	Adult (3oC)	Balanced	57%	(26)
Animalia	Lepidonotothen larseni	Isolated cells (hepatocytes) -max of 6 temperatures	N/A	33	(27)
Animalia	Trematomus eulepidotus	Isolated cells (hepatocytes) -max of 6 temperatures	N/A	38.5	(27)
Animalia	Trematomus pennellii	Isolated cells (hepatocytes) -max of 6 temperatures	N/A	34.2	(27)
Animalia	Trematomus lepidorhinus	Isolated cells (hepatocytes) -max of 6 temperatures	N/A	42	(27)
Animalia	Trematomus bernachii	Isolated cells (hepatocytes) -max of 6 temperatures	N/A	40.4	(27)
Animalia	Artedidraco orianae	Isolated cells (hepatocytes) -max of 6 temperatures	N/A	35.5	(27)
Animalia	Lepidonotothen larseni	Isolated cells (hepatocytes) – min of 6 temperatures	N/A	21.8	(27)
Animalia	Trematomus eulepidotus	Isolated cells (hepatocytes) – min of 6 temperatures	N/A	27.4	(27)
Animalia	Trematomus pennellii	Isolated cells (hepatocytes) – min of 6	N/A	28	(27)

		temperatures			
Animalia	Trematomus lepidorhinus	Isolated cells (hepatocytes) – min of 6 temperatures	N/A	20.9	(27)
Animalia	Trematomus bernachii	Isolated cells (hepatocytes) – min of 6 temperatures	N/A	30.2	(27)
Animalia	Artedidraco orianae	Isolated cells (hepatocytes) – min of 6 temperatures	N/A	26.1	(27)
Animalia	Bufo marinus	Isolated cells	N/A	12%	(28)
Animalia	African catfish (Clarias gariepinus) larvae	Whole animals	Balanced	57%	(29)
Animalia	Rainbow trout	Isolated cells (hepatocytes)	N/A	79.4%	(30)
Animalia	Chrysemys picta bellii	Isolated cells (hepatocytes)	N/A	36%	(31)
Animalia	Glyptonotus antarcticus	Whole animal	N/A	66.4%	(32)
Animalia	Idotea rescata	Whole animal	N/A	21.8%	(32)
Animalia	Goldfish	Hepatocytes	N/A	70.7	(8)
Animalia	Trout	Hepatocytes	N/A	76.7	(8)
Animalia	Human	HepG2 cell line	N/A	8.5	(8)

Y_{ATP} - based estimates

Kingdom	Species	YATP	Culture	Fraction / value	Ref
Bacteria	Lactobacillus caseii	24.3	Glucose limited	68.9	(33)
Bacteria	Aerobacter aerogenes	14	Glucose limited	39.7	(33)
Bacteria	Escherischia coli	10.3	Glucose limited	29.2	(33)
Fungi	Saccharomyces cereviseae	13	Glucose limited	26.8	(33)
Fungi	Candida parapsilosis	12.5	Glucose limited	25.8	(33)
Fungi	Saccahromyces cereviseae	13.4	Rich, Ammonium N source	27.6	(34)
Fungi	Saccahromyces cereviseae	11.9	Rich, Glutamic	24.5	(34)

			acid		
Fungi	Saccahromyces cereviseae	16.3	Rich, mixed amino acids	33.6	(34)
Bacteria	Selenomonas ruminantiurn	10	Optimal	28.3	(35)
Bacteria	Clostridium kluyveri	9	Maximal	25.5	(36)
Bacteria	Escherischia coli	10.3	Anaerobic, glucose limited	29.2	(37)
Bacteria	Lactobacillus plantarum	20	Optimal, aerobic	56.7	(38)
Bacteria	Desulfovibrio vulgaris	12.1	Maximal, H2 + SO4 metabolism	34.3	(39)
Bacteria	Aerobacter cloacae	11.9	Optimal, anaerobic on glucose	33.7	(40)
Bacteria	Escherichia coli	9.4	Rich medium with amino acids	26.6	(40)
Bacteria	Streptococcus diacetilactis	27.7	Complex, rich medium	78.5	(41)
Bacteria	Streptococcus cremoris	17.3	Complex, rich medium	49.0	(41)
Bacteria	Aerobacter aerogenes	10.2	Anaerobic, on glucose	28.9	(42)
Protists	Mixed rumen protozoa	5	Native	12.1	(43)
Fungi	Saccharomyces cereviseae	16	Anaerobic glucose + fatty acids	33.0	(44)
Fungi	Trichoderma QM 9414	10.6	Aerobic on glucose	21.8	(45)
Bacteria	Bdellovibrio bacteriovorus	25.9	Native (inside E.coli cells)	73.4	(46)
Bacteria	Bdellovibrio bacteriovorus	17.9	Native (inside E.coli cells)	50.7	(47)
Archaea	Pyrococcus furiosus	21.17	Maximal	60.0	(48)
Archaea	Pyrococcus furiosus	8.66	Minimal	24.5	(48)
Bacteria	Thermoanaero bacterium saccharolyticu	12	Optimal Fe(III)	34.0	(49)

	m				
Bacteria	Thiobacillus neopolitanus	3	Thiosulfate	8.5	(50)
Bacteria	Zymomonas mobilis	9.9	glucose/ xylose/ acetate	28.1	(51)
Bacteria	Zymomonas mobilis	7.26	glucose/ xylose/ acetate	20.6	(51)
Fungi	Candida tropicalis	4	Fatty acids	8.2	(52)

REFERENCES

1. Lane N, Martin W. The energetics of genome complexity. Nature. 2010;467(7318):929-34.
2. Harold FM. The Vital Force: A Study of Bioenergetics. Gordonsville, VA , USA: W.H. Freeman & Company; 1987.
3. D W Tempest a, Neijssel OM. The Status of YATP and Maintenance Energy as Biologically Interpretable Phenomena. Annual review of microbiology. 1984;38(1):459-513.
4. Mink JW, Blumenschine RJ, Adams DB. Ratio of central nervous system to body metabolism in vertebrates: its constancy and functional basis. American Journal of Physiology - Regulatory, Integrative and Comparative Physiology. 1981;241(3):R203-R12.
5. Solomon S. The coldest march: Scott' fatal antarctic expedition. New Haven, CT, USA: Yale University Press; 2001.
6. BAUCHOP T, ELSDEN SR. The Growth of Micro-organisms in Relation to their Energy Supply. Microbiology. 1960;23(3):457-69.
7. Stouthamer AH, Bettenhaussen C. Utilization of energy for growth and maintenance in continuous and batch cultures of microorganisms. Biochimica et Biophysica Acta (BBA) - Reviews on Bioenergetics. 1973;301(1):53-70.
8. WIESER W, KRUMSCHNABEL G. Hierarchies of ATP-consuming processes: direct compared with indirect measurements, and comparative aspects. Biochemical Journal. 2001;355(2):389-95.
9. Fenchel T, Finlay BJ. Respiration rates in heterotrophic, free-living protozoa. Microbial Ecology. 1983;9(2):99-122.
10. da Silva NA, Bailey JE. Theoretical growth yield estimates for recombinant cells. Biotechnology and Bioengineering. 1986;28(5):741-6.
11. Stouthamer AH. A theoretical study on the amount of ATP required for synthesis of microbial cell material. Antonie Van Leeuwenhoek. 1973;39(1):545-65.
12. Neidhardt FC, Umbarger E. Chemical Composition of Escherichia coli. 1996. In: Escherichia coli and Salmonella: Cellular and Molecular Biology [Internet]. Washington, DC, USA: American Society of Microbiology (ASM) Press
13. Sherman F. Getting started with yeast. In: Christine G, Gerald RF, editors. Methods in Enzymology. Volume 350: Academic Press; 2002. p. 3-41.
14. Yamada EA, Sgarbieri VC. Yeast (Saccharomyces cerevisiae) Protein Concentrate: Preparation, Chemical Composition, and Nutritional and Functional Properties. Journal of Agricultural and Food Chemistry. 2005;53(10):3931-6.
15. Conceição LEC, Dersjant-Li Y, Verreth JAJ. Cost of growth in larval and juvenile African catfish (Clarias gariepinus) in relation to growth rate, food intake and oxygen consumption. Aquaculture. 1998;161(1–4):95-106.
16. Bayne BL. Relations between variable rates of growth, metabolic costs and growth efficiencies in individual Sydney rock oysters (Saccostrea commercialis). Journal of Experimental Marine Biology and Ecology. 2000;251(2):185-203.

17. Jorgensen CB. Metabolic costs of growth and maintenance in the toad, Bufo bufo. Journal of Experimental Biology. 1988;138(1):319-31.
18. Thor P. Relationship between specific dynamic action and protein deposition in calanoid copepods. Journal of Experimental Marine Biology and Ecology. 2000;245(2):171-82.
19. Caton JS, Bauer ML, Hidari H. Metabolic components of energy expenditure in growing beef cattle - review. Asian-Australian Journal of Animal Sciences. 2000;13(5):702 - 10.
20. Hawkins AJS, Widdows J, Bayne BL. The Relevance of Whole-Body Protein Metabolism to Measured Costs of Maintenance and Growth in Mytilus edulis. Physiological zoology. 1989;62(3):745 - 63.
21. Pullar JD, Webster AJF. The energy cost of fat and protein deposition in the rat. British Journal of Nutrition. 1977;37(03):355-63.
22. Bouma TJ, De Visser R, Janssen JHJA, De Kock MJ, Van Leeuwen PH, Lambers H. Respiratory energy requirements and rate of protein turnover in vivo determined by the use of an inhibitor of protein synthesis and a probe to assess its effect. Physiologia Plantarum. 1994;92(4):585-94.
23. Zagdańska B. Respiratory energy demand for protein turnover and ion transport in wheat leaves upon water deficit. Physiologia Plantarum. 1995;95(3):428-36.
24. Hachiya T, Terashima I, Noguchi KO. Increase in respiratory cost at high growth temperature is attributed to high protein turnover cost in Petunia × hybrida petals. Plant, Cell & Environment. 2007;30(10):1269-83.
25. Pace DA, Manahan DT. Cost of Protein Synthesis and Energy Allocation During Development of Antarctic Sea Urchin Embryos and Larvae. The Biological Bulletin. 2007;212(2):115-29.
26. Bowgen AD, Fraser KPP, Peck LS, Clarke A. Energetic cost of synthesizing proteins in Antarctic limpet, Nacella concinna (Strebel, 1908), is not temperature dependent. American Journal of Physiology - Regulatory, Integrative and Comparative Physiology. 2007;292(6):R2266-R74.
27. Mark FC, Hirse T, Pörtner HO. Thermal sensitivity of cellular energy budgets in some Antarctic fish hepatocytes. Polar Biology. 2005;28(11):805-14.
28. Fuery CJ, Withers PC, Guppy M. Protein Synthesis in the Liver of Bufo marinus: Cost and Contribution to Oxygen Consumption. Comparative Biochemistry and Physiology Part A: Molecular & Integrative Physiology. 1998;119(2):459-67.
29. Conceição LEC, Houlihan DF, Verreth JAJ. Fast growth, protein turnover and costs of protein metabolism in yolk-sac larvae of the African catfish (Clarias gariepinus). Fish Physiology and Biochemistry. 1997;16(4):291-302.
30. Pannevis MC, Houlihan DF. The energetic cost of protein synthesis in isolated hepatocytes of rainbow trout (Oncorhynchus mykiss). 162. 1992;5(393 - 400).
31. Land SC, Buck LT, Hochachka PW. Response of protein synthesis to anoxia and recovery in anoxia-tolerant hepatocytes. American Journal of Physiology - Regulatory, Integrative and Comparative Physiology. 1993;265(1):R41-R8.
32. Whiteley NM, Taylor EW, el Haj AJ. A comparison of the metabolic cost of protein synthesis in stenothermal and eurythermal isopod crustaceans. American Journal of Physiology - Regulatory, Integrative and Comparative Physiology. 1996;271(5):R1295-R303.
33. Russell JB, Cook GM. Energetics of bacterial growth: balance of anabolic and catabolic reactions. Microbiological Reviews. 1995;59(1):48-62.
34. Albers E, Larsson C, Lidén G, Niklasson C, Gustafsson L. Influence of the nitrogen source on Saccharomyces cerevisiae anaerobic growth and product formation. Applied and Environmental Microbiology. 1996;62(9):3187-95.
35. HOBSON PN, SUMMERS R. ATP Pool and Growth Yield in Selenomonas ruminantium. Microbiology. 1972;70(2):351-60.
36. Thauer RK, Jungermann K, Henninger H, Wenning J, Decker K. The Energy Metabolism of Clostridium kluyveri. European Journal of Biochemistry. 1968;4(2):173-80.

37. Hempfling WP, Mainzer SE. Effects of varying the carbon source limiting growth on yield and maintenance characteristics of Escherichia coli in continuous culture. Journal of Bacteriology. 1975;123(3):1076-87.
38. Giraud E, Lelong B, Raimbault M. Influence of pH and initial lactate concentration on the growth of Lactobacillus plantarum. Applied Microbiology and Biotechnology. 1991;36(1):96-9.
39. Badziong W, Thauer RK. Growth yields and growth rates of Desulfovibrio vulgaris (Marburg) growing on hydrogen plus sulfate and hydrogen plus thiosulfate as the sole energy sources. Archives of Microbiology. 1978;117(2):209-14.
40. Hernandez E, Johnson MJ. Anaerobic Growth Yields of Aerobacter cloacae and Escherichia coli. Journal of Bacteriology. 1967;94(4):991-5.
41. Brown WV, Collins EB. End products and fermentation balances for lactic streptococci grown aerobically on low concentrations of glucose. Applied and Environmental Microbiology. 1977;33(1):38-42.
42. Hadjipetrou LP, Gerrits JP, Teulings FAG, Stouthamer AH. Relation between Energy Production and Growth of Aerobacter aerogenes. Microbiology. 1964;36(1):139-50.
43. Nolan J. Implications of protozoa and fungi for the protein nutrition of ruminants. The Rôles of Protozoa and Fungi in Ruminant Digestion: Penambul Books, Armidale, NSW; 1989. p. 211-21.
44. VERDUYN C, POSTMA E, SCHEFFERS WA, VAN DIJKEN JP. Energetics of Saccharomyces Cerevisiae in Anaerobic Glucose-Limited Chemostat Cultures. Microbiology. 1990;136(3):405-12.
45. Harima T, Humphrey AE. Estimation of Trichoderma QM 9414 biomass and growth rate by indirect means. Biotechnology and Bioengineering. 1980;22(4):821-31.
46. Rittenberg SC, Hespell RB. Energy efficiency of intraperiplasmic growth of Bdellovibrio bacteriovorus. Journal of Bacteriology. 1975;121(3):1158-65.
47. Hespell RB. Intraperiplasmic Growth of Bdellovibrio bacteriovorus on Heat-Treated Escherichia coli. Journal of Bacteriology. 1978;133(3):1156-62.
48. Kengen SM, Stams AJM. Growth and energy conservation in batch cultures of Pyrococcus furiosus. FEMS Microbiology Letters. 1994;117(3):305-9.
49. Wiegel J, Hanel J, Aygen K. Chemolithoautotrophic thermophilic iron (III)-reducer. In: Ljungdahl LG, Adams MW, Barton LL, Ferry JG, Johnson MK, editors. Biochemistry and Physiology of Anaerobic Bacteria: Springer; 2003. p. 235-51.
50. Kelly D. Energetics of chemolithotrophs: Academic Press, San Diego; 2012.
51. Jeon YJ, Svenson CJ, Joachimsthal EL, Rogers PL. Kinetic analysis of ethanol production by an acetate-resistant strain of recombinant Zymomonas mobilis. Biotechnology Letters. 2002;24(10):819-24.
52. Gallo M, Azoulay E. YATP value in Candida tropicalis grown on n-alkanes, fatty acids, and acetate. Biotechnology and Bioengineering. 1975;17(12):1705-15.
