# Supplementary material for: Evolution of default genetic control mechanisms
Source: PLoS One. 2021 May 13;16(5):e0251568. doi: 10.1371/journal.pone.0251568 (PMC8118313; doi:10.1371/journal.pone.0251568)
Supplement: Attachment — Submitted filename: Response to reviewers V 3.0.docx [file pone.0251568.s003.docx]

We are grateful to the reviewer for their detailed comments. Their comments below are in **bold,** our responses are in plain test. Line numbers refer to the Track Changes version of the revised paper.

**First of all, there is probably a problem in the fundamental definition of “default off” logic. In Line 115, the authors stated, “We argue that this ‘Default off’ logic is more efficient if the majority of the genome is silent.” Is this tautology? I think “the majority of the genome is silent“ is just synonymous with the “default off” logic.**

We apologise for not making this clearer. The concept of a ‘default off’ logic is that the genome controls are structured such that it is ‘easier’ for a gene to be inactive than to be active. Specifically, more metabolic energy needs to be expended to turn a gene on in a ‘default off’ genome. This does not mean that genes *are* inactive. The entire genome could be active at the same time in a ‘default off’ genome – that would just take a lot more metabolic energy to achieve than in a ‘default on’ genome.

To use an analogy, it requires less energy for a human to be sitting down than running. Our ‘default’ state is sitting down and (relatively) inactive. That is not to say that marathons, where everyone is running, cannot happen, just that they take energy to make happen.

We have made this point explicitly in lines 118 – 124., and added some additional text to line 97 – 99 and 396

**Second, the assumptions about “environments” is not very clear. And the underlying biological reasoning is not clear as well. In Fig 3A-B, the authors demonstrated a simple case under a certain environment. Then suddenly in Fig 3C, there are three environments. Both the purpose of increasing the number of environments and the biological scenarios to depict by the increase are not very clear.**

We have revised the text around this substantially, adding new explanation in lines 204 - 215. In essence, the complexity of the environment to which a genotype adapts could be a single complex environment (say, a rainforest) or several simpler environments which alternate (say, a tidal pool at high and low tide). We have tried to make the rationale for including both in our model clearer.

Figure 3C was included to illustrate that the model does adapt when presented with multiple alternating environments, but if the reviewer thinks this is confusing and does not help understanding, we can remove this illustration.

**Especially, the author attempted to test the hypothesis on the environmental complexity and the evolution of the default mode. Presumably, the optimal goal of gene control is to express different certain sets of genes at different time or at different habitats.**

This is our expectation, yes, and the model is built around this idea

**That could be modeled by fluctuations with these “environments.” But in this study, it seems that the environmental complexity only represents the number of possible environments existed for matching instead of frequent alternations among possible environments with time. I am really curious if using the fluctuating environments would lead to different conclusions.**

We also were interested in this! We have expanded our discussion of the environmental parameters in lines 202 – 213 and 375 - 380. We have also addressed this with some additional analysis, which shows that whether the environment fluctuates or not does affect the correlation of environment with default genetic mode. However it suggests that fluctuating environments are weakly correlated with ‘default on’ genetics, which is not what we would expect. We discuss this briefly (lines 369 – 373). We have not explored enough of the parameter space to see if the rate of fluctuation or the number of different environments has an effect.

**Also, I have doubts on the measurements for “default off” and “default on” logic. While I understand why the shorter elements have a higher likelihood to be active in the model, that does not seem to reflect the real biology of gene regulation/control. The activity of elements is usually determined by the matching specificity between the sequence of regulatory elements and the regulatory molecules in the environments. It is not easy to rationale the biological meaning on the role of length in the simulation here.**

**After all, I feel the evolution of "default off" logic results from the gains of negative elements with strong specificity instead of the decreasing specificity of negative elements for spurious interactions. Of course, whether this is true requires more empirical data.**

The reviewer brings up a very good point. In Eukaryotes controls with very little sequence specificity (analogous to short sequences in our model) are common in the folding and compaction of chromatin, e.g. the binding of H2A/H2B histone pair to nucleosomes, which require energy to remove but are essentially sequence-agnostic. This is accurately captured by our model. However other controls are likely to be sequence specific.

We have therefore re-analysed the model runs to ask whether negative elements that interact with ‘coding’ sequences present in the same cell also show correlations with genome or environmental factors (lines 309 – 312, 336 – 350 and new columns in Table 1). This explicitly probes the regulatory sequence + regulator pair that the reviewer mentions. We have added this analysis to Table 1. The result show very similar correlations to the existing model.

**Finally, the author stated they found a “striking” correlation between the fraction of expressed genes and the tendency of “default on” logic. But if the “default on” logic suggests that more positive elements are active, isn’t the higher fraction of expressed genes just a natural outcome of that?**

As above, ‘default on’ means that it is ‘easier’ to turn a gene on, not that it necessarily is on under any selective regime. We agree that the result is ‘obvious’, but to our knowledge it has not been pointed out before that the nature of the gene control architecture of eukaryotes is best suited to a genome that is mostly not expressed at any one time.

**Minor comments:**

**In Fig 2a, why the arrow of transcription is from the phenotype to genotype? It should be opposite.**

Correct – our apologies! We have corrected this.

**In Fig 2b, what “ve” means should be explained in more details in the text around page 6.**

We have added some text on this, in lines 156 - 157

**There is an error on reference format in Line 193.**

We have corrected this

**I appreciate the authors have considered the percentage to full adaptation and illustrated cases of adaptation very clearly. But I would further encourage the authors to also run the simulation without any fitness differences in the model at all and see if non-adaptation parameters in the models (e.g. mutation rates and choice of measurements) are sufficient to create the correlations (a negative control of the methodology).**

This is a good idea, and we thank the reviewer for it. We have now carried out a set of ‘control’ model run where replication was uncoupled from fitness, and have included details of these in the revised paper, in a new section 3. (lines 423 - 441). This exercise did uncover a bias in one measure, which explains why this meaure was more poorly correlated with outcomes.
