# Supplementary material for: Evolution of default genetic control mechanisms
Source: PLoS One. 2021 May 13;16(5):e0251568. doi: 10.1371/journal.pone.0251568 (PMC8118313; doi:10.1371/journal.pone.0251568)
Supplement: Attachment — Submitted filename: PLoS reply to reviewer round 2.docx [file pone.0251568.s004.docx]

**Reviewer #1: All of my previous questions are well addressed. But I have new questions for the newly added section of ‘Existing genome’ measures. In Line 343~344, the statement suggests that larger E_g means a more likely "default-on" mode. This definition is not consistent with the definitions of R_a and R_m, whose larger values suggest a more likely "default-off" mode (Line 315~317 + Line 326~328). Then, with that inconsistency, in the table 1, why do they tend to show the same sign of correlation coefficients for various model parameters? I hope the authors can check the definitions of these measurements, make these measurements more consistent with each other if possible, and clarify whether these different measures lead to similar correlations in the modified manuscript.**

We understand the reviewer’s confusion, and apologise for not explaining this more clearly. In all measures, a value of <1 is associated with a ‘default off’ mode. In the ‘new gene’ modes, regulatory element length is inversely related to the probability that that element is active. So length(negative elements)/length(positive) elements is <1 if the length of the negative elements is shorter than the length of the positive elements, and hence the probability of the negative elements being active is larger than the probability of the positive elements being active. For the ‘existing genome’ measures we are measuring actual activity, not the chance of activity through matching to a random sequence. So if positive/negative <1, more negative elements are active than positive ones.

This was not made clear in the text, and we have added some text to try to remedy this. We hope it is better explained now.

The wording in lines 315-317 and 326-328 was also ambiguous, and suggested that high values of Ra and Rm meant ‘default off’. In Table 1 it was correctly stated that for all measures <1 implied ‘default off’. We have corrected this wording.

**One typo in the manuscript: L316- two equal signs in the equation?**

Thank you, yes this was an error, now corrected.

We have also added a reference to the introduction to a recent Nature paper on proteins in chromatin structure in Yeast, which has some relevance.
